# Training for mental health professionals in responding to experienced and anticipated mental health related discrimination (READ-MH): protocol for an international multisite feasibility study

**DOI:** 10.21203/rs.3.rs-1466318/v1

**Published:** 2022-03-28

**Authors:** Claire Henderson, Uta Ouali, Ioannis Bakolis, Nada Berbeche, Kalpana Bhattarai, Elaine Brohan, Anish Cherian, Eshetu Girma, Petra C Gronholm, Dristy Gurung, Charlotte Hanlon, Sudha Kallakuri, Amanpreet Kaur, Bezawit Ketema, Heidi Lempp, Jie Li, Santosh Loganathan, Pallab K. Maulik, Gurucharan Mendon, Tesfahun Mulatu, Ning Ma, Renee Romeo, Rahul Kodihalli Venkatesh, Yosra Zgueb, Wufang Zhang, Graham Thornicroft

**Affiliations:** King’s College London Institute of Psychiatry Psychology and Neuroscience; Razi University; King’s College London Institute of Psychiatry Psychology and Neuroscience; University of Tunis: Universite de Tunis; TPO Nepal: Transcultural Psychosocial Organization Nepal; King’s College London Institute of Psychiatry Psychology and Neuroscience; NIMHANS: National Institute of Mental Health and Neuro Sciences; Addis Ababa University; King’s College London Institute of Psychiatry Psychology and Neuroscience; TPO Nepal: Transcultural Psychosocial Organization Nepal; King’s College London Institute of Psychiatry Psychology and Neuroscience; The George Institute for Global Health; The George Institute for Global Health; Addis Ababa University School of Public Health; King’s College London; Affiliated Brain Hospital of Guangzhou Medical University: Guangzhou Huiai Hospital; NIMHANS: National Institute of Mental Health and Neuro Sciences; The George Institute for Global Health; NIMHANS: National Institute of Mental Health and Neuro Sciences; Addis Ababa University Department of Community Health: Addis Ababa University School of Public Health; Peking University Institute of Mental Health: Peking University Sixth Hospital; King’s College London Institute of Psychiatry Psychology and Neuroscience; NIMHANS: National Institute of Mental Health and Neuro Sciences; Razi Hospital: Hopital Razi; Peking University Institute of Mental Health: Peking University Sixth Hospital; King’s College London Institute of Psychiatry Psychology and Neuroscience

**Keywords:** stigma, discrimination, training, health professionals, mental health care, objective structured clinical examination, health advocacy

## Abstract

**Background:**

Mental health and other health professionals working in mental health care may contribute to the experiences of stigma and discrimination among mental health service users, but can also help reduce the impact of stigma on service users. However the few studies of interventions to equip such professionals to be anti-stigma agents those took place in High-Income Countries. This study assesses the feasibility, potential effectiveness and costs of Responding to Experienced and Anticipated Discrimination training for health professionals working in mental health care (READ-MH) across Low-and Middle-Income Countries (LMICs).

**Methods:**

This is an uncontrolled pre-post mixed methods feasibility study of READ-MH training at seven sites across five LMICs (China, Ethiopia, India, Nepal, and Tunisia). Outcome measures: knowledge based on course content; attitudes to working to address the impact of stigma on service users; and skills in responding constructively to service users’ reports of discrimination. The training draws upon the evidence bases for stigma reduction, health advocacy and medical education and is tailored to sites through situational analyses. Its content, delivery methods and intensity were agreed through a consensus exercise with site research teams. READ-MH will be delivered to health professionals working in mental health care immediately after baseline data collection; outcome measures will be collected post-training and three months post-baseline, followed by qualitative data collection. Fidelity will be rated during delivery of READ-MH, and data on training costs will be collected. Quantitative data will be assessed using generalised linear mixed models. Qualitative data will be evaluated by thematic analysis to identify feedback about the training methods and content, including the implementability of the knowledge and skills learned. Pooled and site-specific training costs per trainee and per session will be reported.

**Conclusions:**

The training development used a participatory and contextualized approach. Evaluation design strengths include the diversity of settings; the use of mixed methods; the use of a skills-based measure; and knowledge and attitude measures aligned to the target population and training. Limitations are the uncertain generalisability of skills performance to routine care, and the impact of COVID-19 restrictions at several sites limiting qualitative data collection for situational analyses.

## Background

In 1998, The Lancet published an essay by Norman Sartorius, then President of the World Psychiatric Association, entitled ‘Stigma: what can psychiatrists do about it?’ (Sartorius, 1998). Focusing particularly on schizophrenia, he recommended that psychiatrists: 1) expand the focus of clinical work beyond symptom reduction to improving quality of life; 2) reflect on and try to improve their own attitudes by updating their clinical knowledge and learning from those using their services and their families about the impact of the illness and of stigma on them; 3) monitor for discrimination and expand their role to include advocacy; and 4) learn from others about how stigma and discrimination can be reduced.

Over twenty years later, it is now timely to consider progress against these recommendations. Outcomes other than clinical ones are increasingly used in research and routine practice, and the concept of personal recovery has had considerable impact on mental health policy and practice in many countries (Le Boutillier et al., 2011). Further, continuing professional development is included in the requirements for license renewals and revalidation for many professionals. Reflective practice is used extensively in undergraduate and postgraduate training (Schutz, 2007) which in theory provides scope for examining one’s own attitudes to people with mental illness. While the extent to which this occurs is unknown, stigma among health professionals, including mental health professionals is an increasing focus of research (Henderson et al., 2014).

Although mental health professionals have more knowledge of mental illness compared to health professionals of other specialties, they are nevertheless exposed to negative cultural stereotypes prior to professional education (Schulze, 2007). Indeed they differ little from the general population in terms of desire for social distance, but consistently show less socially restrictive attitudes (except regarding coercion into treatment) and are more supportive of the civil rights of people with mental illness (Lauber, Anthony, Ajdacic-Gross, & Rössler, 2004; Magliano et al., 2004; Vibha, Saddichha, & Kumar, 2008).

The frequency of discriminatory behaviours reported by service users in mental health care settings ranges from 16% to 44% (Gabbidon et al., 2014; C. Thornicroft, Wyllie, Thornicroft, & Mehta, 2014). Qualitative research in England found that service users described a lack of support and a tendency towards overprotectiveness by mental health service providers (Hamilton et al., 2016), while in Mexico service users described cold and distant treatment and a lack of clear explanations provided by psychiatrists, a focus on psychopathology/medication and lack of interest in their personal history (Lagunes-Cordoba et al., 2021). By means of their particular relationship with the service user, mental health care professionals can contribute to exacerbating or mitigating self-stigma (Wang, Link, Corrigan, Davidson, & Flanagan, 2018). This form of stigma encompasses negative beliefs about the self, based on shame, the acceptance of mental illness stereotypes, a sense of alienation from others, and consequent low self-esteem and mood. It prevents people from seeking healthcare, employment and social opportunities (Corrigan & Angermeyer, 2012).

However, less stigmatisation of patients by more experienced mental health professionals was observed in several surveys (Jorm, Korten, Jacomb, Christensen, & Henderson, 1999; Lauber, Nordt, Braunschweig, & Rossler, 2006; Linden & Kavanagh, 2012). This may be attributed to a better capacity to preventing burnout and the loss of empathy associated with burnout, more observations of personal recovery in patients, more personal or family experience of mental illness, and/or more experience in overriding stereotypes (Henderson et al., 2014).

While the evidence above suggests mental health professionals as a target for a stigma reduction intervention, any such intervention need also to take into consideration the mental health professionals’ potential role as an anti-stigma change agent (Deb et al., 2019). Previous articles have acknowledged the potential impact that physicians’ advocacy could have in reducing discrimination (Arboleda-Flórez & Stuart, 2012; Thornicroft et al., 2010; Ungar et al., 2016), agreeing professionals could champion anti-stigma efforts, including much needed structural changes as health care quality improvement and policy change. The extent to which stigma reduces mental health professionals’ ability to provide effective care is obvious to them and can contribute to burnout and demotivation.

At a policy and service level, stigma contributes to restrictive mental health legislations, the poor coverage of mental health education in university curricula for health professionals, unequal allocation of health resources with low budgets attributed to mental health care compared to physical health care resulting in poor mental health care resources (Saxena, Thornicroft, Knapp, & Whiteford, 2007), in over-reliance on institutional care, and in limited access to physical health care (Perry, Lawrence, & Henderson, 2020). Mental health professionals notice service users’ barriers to seeking and engaging with treatment, obstacles to rehabilitation due to discrimination in employment and within social networks, reluctance to pursue economic and social opportunities due to the anticipation of discrimination, and negative self-evaluation due to internalised stigma (Zaske, Freimuller, Wolwer, & Gaebel, 2014) However, there is little evidence that advocacy and effective stigma reduction methods have been incorporated into the role of psychiatrists or other mental health professionals, based on reviews of stigma reduction interventions (Mehta et al., 2015; G. Thornicroft et al., 2016). As a result, how anti-stigma advocacy can be incorporated by mental health professionals is not yet clear. We therefore need to return to Sartorius’s recommendation to learn from others.

In other fields of medicine and especially that of primary care, there is an increasing focus on physicians’ social accountability and advocacy. Across North America, for example, several organizations have expressed a pressing need for advocacy training in medical education (ACGME, 2007; Frank et al., 2015; Shaws et al., 2017). The Royal College of Physicians and Surgeons of Canada published a CanMEDS Physician Competency Framework, introducing health advocacy as one of six main competencies which medical education programs need to address (CanMEDS: Better Standards, Better Physicians, Better Care, 2011). The role of health advocate is understood as the skill to “determine and understand needs, speak on behalf of others when required, and support the mobilization of resources to effect change” (Frank, 2015). The CanMEDS competencies have been adopted in a number of other countries including an adaptation to the Ethiopian context (EthioMEDS) for psychiatry training (Alem, Pain, Araya, & Hodges, 2010), where psychiatrists are likely to be called upon to develop policy, services, training and to join with service users to advocate for better services. However, there is a need to introduce the concept of anti-stigma agency to other cadres and to combine it with stigma reduction.

A recent systematic review (Guerrero) of the feasibility and effectiveness of training in health advocacy, anti-stigma competency or related skills for health professionals retrieved 39 studies, three of which reported interventions for mental health care professionals (Griffith & Kohrt, 2016; Li et al., 2019; Sheely-Moore & Kooyman, 2011; Zaske et al., 2014). Program content varied widely; some covered mainly interpersonal stigma reduction, others focusing on social determinants of health, and some included health advocacy at the structural level. The authors found some evidence for their effectiveness, however, it proved difficult to compare effectiveness across programmes given the wide variety in content, duration, teaching methods and outcome measures. The majority of studies were carried out in High Income Countries (HICs), therefore it is difficult to extrapolate feasibility to Low- and Middle-Income Countries (LMICs) where the mix of professionals and hence the roles they carry out is often different to that of HICs. The authors conclude that to maximise its relevance to the communities served, any intervention for mental healthcare professionals needs to: link to the professionals’ roles; be developed following a situational analysis; and include local people with lived experience of mental health related stigma in the delivery (Experts by Experience). Training needs to use interactive delivery methods and initial piloting; and evaluation should examine behavioural change.

We therefore designed Responding to Experienced and Anticipated Discrimination training for health professionals working in mental health services (READ-MH). These may be mental health professionals or health professionals who do not have professional training in mental health but are working in mental health services. Further, our target service setting is specialist mental health services rather than primary care or any other setting. READ-MH was developed based on: a previous training for medical students (Deb et al., 2019; Potts); the findings of the above review; situational analysis at the seven sites of the INDIGO Partnership programme (Gronholm) comprising desk reviews and qualitative interviews and focus groups with stakeholders on mental health related stigma; and consensus development regarding the delivery format, content and teaching methods among the INDIGO Partnership research team based on data from the studies included in the systematic reviews. The present study aims to assess the feasibility, potential effectiveness and costs of the READ-MH training at these sites.

## Methods/design

This is a pre-post feasibility study at seven LMIC sites using mixed methods. READ-MH will be delivered to health professionals working in mental health care immediately after baseline data collection; the quantitative follow up data will be collected post training and at three months post baseline, followed by qualitative data collection. Fidelity will be rated during READ-MH delivery. In addition, data on the costs of the training will be collected. There is no control group as this is not required to address aims related to feasibility, and the sample size is not designed to determine effectiveness.

### Setting

All seven sites across five countries (China, Ethiopia, India, Nepal, and Tunisia) of the UK Medical Research Council funded INDIGO-Partnership research group will take part in the study. Given that sites have variable provision of mental health care and staffing, sample sizes and targeted professionals vary by site (see Table 1).

### Participants and Recruitment

Eligible mental health professionals or health professionals will be working currently in the mental health field at each site. A participant information sheet will be distributed by the site researchers to eligible professionals at least 24 hours before the first session, and written informed consent will be sought at the start of the first session. Individual written consent will be ensured through the availability of multiple members of the research team who will answer individual professionals’ queries. It will be made clear to professionals that non-participation will not be penalised by the professional’s supervisor.

### Measures

#### Outcome measures

Knowledge will be assessed through a structured questionnaire in the form of a quiz based on the content of the training with input from all sites. The quiz will cover (i) knowledge about sources of stigma; (ii) the impact of stigma including in the context of health care; and (iii) how mental health professionals can reduce this impact.

Attitudes of mental health professionals to working to address the impact of stigma on service users will be measured through the ASTAD (Attitudes to addressing stigma and discrimination scale). This scale was created by rewording an existing scale, the Short Alcohol and Alcohol Problems Perception Questionnaire (SAAPPQ) (Anderson & Clement, 1987). The original SAAPPQ assesses health professionals’ attitudes to working with people with alcohol problems. It has two items contributing to each of five domains (Role adequacy, Role legitimacy, Motivation, Work satisfaction and Task-specific self-esteem) and two subscales: Role security (the sum of role Adequacy and Role legitimacy) and Therapeutic commitment (the sum of Motivation, Work Satisfaction and Task-specific Self-esteem). In the British validation study, the SAAPPQ showed good correlation with the extent of postgraduate training in addiction and the frequency at which GPs initiated discussions about alcohol use during consultations (Anderson & Clement, 1987). By retaining the questions and changing the wording from working with people with alcohol problems to working to reduce stigma and discrimination, the ASTAD retains the structure of the SAAPPQ.

Behaviour and communication skills will be assessed through an objective structured clinical examination (OSCE). These are widely used in medical education and comprise scenarios in which the student/trainee interacts with a simulated patient in the presence of an examiner (Khan, Ramachandran, Gaunt, & Pushkar, 2013). The scenario for READ-MH comprises a simulated mental health service user reporting experienced and anticipated discrimination and who is faced with a disclosure decision regarding their mental illness, either in the context of marriage or employment. The OSCE-scenario was developed in according with the INDIGO Partnership-implementing sites to reflect typical interactions/ problems of discrimination/stigma at the sites.

Participating simulated service users will be given a briefing sheet to standardise the scenario and their responses to participating professionals’ questions.

The professional will be assessed by both the simulated patient and the observer on: (i) their responses aimed to reduce the impact of stigma, including acknowledging the problem, showing an empathic attitude, exploring the patient’s concerns, and identifying important considerations to help the patient make a decision informed by their own values and relevant clinical information; (ii) stigmatising behaviours such as ignoring the simulated patient’s concerns; dismissing/not believing their report of discrimination; endorsing the discriminatory behaviour reported; attributing responses to anticipated discrimination to laziness or incompetence; or telling the patient whether to make a disclosure.

Guidance for the OSCE raters and simulated patients for their assessment will be adapted from existing communication skills OSCEs used at King’s College London; the OSCE developed for the study of READ for medical students, and from the ENhancing Assessment of Common Therapeutic factors (ENACT) therapist competence training rating scale (Kohrt et al., 2015). This guidance will be transcribed into a standardized marking scheme describing the assessment process to increase reliability and comparability across sites. Marking will reflect the formative nature of the OSCE.

#### Implementation measures

Quantitative process measures will include: (i) attendance records for participants and (ii) a fidelity checklist to record delivery of and participants’ active participation in the sessions. Items on delivery will cover the use of those evidence-based teaching methods chosen. Each item in the checklist will be scored 0= not achieved; 1= partially achieved; or 2= achieved in full, with guidance for the anchor points. Qualitative process data will include feedback on the training immediately post training, and feedback on any impact on the professionals and their practice at three months.

### Intervention

We describe READ-MH using the TIDieR checklist (Hoffmann et al., 2014). The aims of READ-MH are to increase the ability of professionals working in mental health care to:

1. recognise their own stigma and minimise the effects on patients, carers, students and trainees in health professions, and colleagues
2. identify and respond constructively to patients’ reports of and anticipated discrimination and self-stigma
3. address interpersonal discrimination and foster advocacy at the individual, family and service levels

The READ-MH manual was developed with input from each site from its inception to adapt the content and delivery to the cultural and social specificities of each site, following a cultural adaptation matrix. This matrix was established through situation analysis comprising a desk review on mental health related stigma in the region and qualitative research with local healthcare staff, service users and carers. The manual describes each module including methods of delivery (role play, facilitated group discussion, testimony from the Expert by Experience, etc.), provides a slide pack and gives specific examples of adaptation to each site. The manual also contains guidance on training and supporting the Expert by Experience, ground rules for the interaction of mental health professionals with the Expert by Experience and a safety protocol in case the Expert by Experience requires support during or after the delivery of READ-MH.

READ-MH training will be provided by research team members at each site who are themselves mental health professionals together with Experts by Experience. Delivery will be to mental health professionals in groups of no more than 12 participants. Training will be delivered in five modules, which allow for flexibility across sites: all in one time, shorter or longer duration of training (4 to 8 hours). The different modules, their respective content and teaching methods are shown in Table 2. Training will be in person unless COVID-19 restrictions require online training and internet connections are sufficiently reliable.

Experts by Experience will play an important role in delivering the training. Each site research team will provide training to Experts by Experience to prepare them to give personal testimony. The training will be based on previous work at one of the sites (Rai et al., 2018) and on training for Time to Change Champions (https://www.time-to-change.org.uk/take-action/resources-champions). The contribution of the Expert by Experience to the training will include: (i) descriptions of the illness and personal recovery process; (ii) experiences of stigma and discrimination in the community and in mental health care services; and (iii) coping with stigma including with its internalization, its anticipation, and with experiences of discrimination and how they were overcome.

It is possible that Experts by Experience may find that delivering the training acts as a stressor that affects their mental health, or that a mental health professional behaves in a way they find upsetting during the training. To minimise this risk, ground rules for the mental health professionals will be established. These will include: not asking questions used for clinical assessment of the Expert by Experience’s mental state; not dismissing the expert’s experience; and not interrupting them. We will also use a safety protocol for Experts by Experience informed by the anti-stigma programme for Time to Change England to ensure they are well-supported. This will include avoidance of training sessions directly before times when support is not accessible, such as weekends; debriefing after each session; and a contact number for experts to call a clinical member of the research team if they wish to debrief at a subsequent time.

### Procedures

#### Quantitative data

Baseline data will be collected immediately before the first training session starts (see Table 3). The OSCE will be administered first, to prevent an influence on behaviour during the OSCE of considering responses to the knowledge quiz and ASTAD. Each OSCE will be rated by one of the trainers or another member of the research team. Afterwards, participants will be given the ASTAD and knowledge quiz for self-completion.

The fidelity checklist and attendance record will be completed by a member of the research team at each session. Immediate post training data collection will occur at the end of the training and will follow the same order as for baseline data collection. Results of the two OSCEs will be provided once all immediate post training follow up data have been collected. At three months from the baseline, participants will be given or sent the knowledge quiz and ASTAD to self-complete and return.

#### Qualitative data

At each site, all participants in each group will be invited to attend a focus group held once they complete the OSCEs and outcome assessments immediately after the training. The topic guide will cover their perceived relevance and usefulness of the training; aspects they thought worked best versus less well; parts of the training that could be improved; and barriers/facilitators to application in practice.

At three months follow up, after completion of the outcome assessments, all participants will be invited to take part in focus groups or individual interviews. The topic guide will cover any perceived impact on practice and if relevant education; barriers/facilitators to application in practice; and experiences of application to practice/education. The choice of interviews versus focus groups will be made by each site research team. Interviews and focus groups will be audio recorded and transcribed verbatim by members of the research site teams.

Table 3 gives an overview of all quantitative and qualitative assessments at the different time points.

#### Health Economics

An economic research question concerns costs: what is the cost to introduce READ-MH? Therefore, in this study we will examine the cost of READ-MH training, including the comparative costs of such an intervention across different geographical sites. All sites will take part in an interview designed to get a description of the training to be implemented in their site and to obtain their best estimates of the resources required. After the interview, an Excel spreadsheet will be sent to each site, requesting the relevant data to facilitate the derivation of site-specific READ-MH training costs.

Data on time spent by various individuals in training will be measured prospectively by the research teams at each site. Attendance and data on time spent at training sessions will also be measured prospectively by the research team at each site.

All costs will be collected in local currencies and converted to US$ using Purchasing Power Parity (PPP) and local currency unit (LCU) exchange rates for the most recent year. Training sessions will be costed for the trainers and Experts by Experience using per diem payments and will include preparation time for the sessions. Direct training-related expenses will be identified such as: training venue room hire, IT equipment, and training materials. These costs will be obtained retrospectively from the research team immediately after training. Other training-related expenses will cover: accommodation, travel and subsistence for trainers and Experts by Experience if not included in the stipend; translators and subsistence for translators; trainee stipend and subsistence for trainees if not covered in the stipend; driver time payment; petrol/diesel; refreshments and catering expenses for the trainee and any other person accompanying the trainer or the trainee to assist with child care.

#### Data analysis

Quantitative data will be assessed using generalised linear mixed models depending on the distribution of the outcome (continuous, binary). Descriptive statistics of quantitative outcome data (OSCE score, ASTAD, knowledge quiz) will be provided. The impact of the training on outcome measures will be analysed comparing the OSCE scores, ASTAD and knowledge quiz at baseline, post-training and 3 months post training. A three-level hierarchical model will be employed and all time points will be included as repeated measures in the model to improve power and account for clustering of observations at site levels.

Qualitative data will be analysed by thematic analysis (Braun & Clarke, 2006) of the interview and focus group transcripts using NVivo qualitative computer software. Coding and translation of illustrative accounts will be undertaken by research staff fluent in the language of the transcript and English so that a coding framework in English based on data from all sites can be created. A combined deductive and inductive approach will be applied to identify and compare themes across sites in terms of responses to the training methods and content, including the implementability of the knowledge and skills learned including barriers encountered.

For health economics analysis, the training cost will be summed and weighted by the number of individuals trained and the number of training sessions to derive a total cost per trainee and a total cost per session. Pooled and site-specific training costs will be reported. We will describe the total and component training costs presenting median, mean, standard deviations, and ranges.

One way sensitivity analyses will be undertaken to explore the sensitivity of the results to changes in assumptions.

## Discussion

This study has several strengths in terms of the intervention and evaluation designs. The training draws upon the evidence bases for stigma reduction, health advocacy and medical education. In addition, the training is tailored from the outset to each site through the selection of locally relevant data from the situational analyses. Further, the content, delivery methods and intensity of the training have been agreed through a consensus exercise with site research teams to enhance feasibility. Strengths of the evaluation design include the diversity of settings, improving generalisability of the results; the use of a mixed methods design; the use of a behavioural outcome measure in the form of the OSCE; and the use of knowledge and attitude measures appropriate for the target population and the training. Key limitations are the uncertain generalisability of OSCE performance to routine care, and our inability to assess effectiveness or cost-effectiveness in relation to patient outcomes at this stage. Finally, the existence of COVID-19 restrictions at several sites limited the extent of qualitative data collection for the situational analyses to inform the development of READ-MH.

## Figures and Tables

**Table 1.** Study settings

Site	Location for training	Target professionals	Target sample and size
Beijing, China	Haidian, Chaoyang, Xicheng, district, Beijing	Psychiatry residents, psychiatrists, psychiatric nurses, social workers working in Peking university sixth hospital and district mental health hospitals	30 mental health professionals
Guangzhou, China	Guangzhou, China	Psychiatry Residents, Primary Health Care Providers (General Practitioners, Community Psychiatric Nurses, etc.)	20 mental health professionals
Ethiopia	Sodo, South Sodo, Misrak Meskan and Meskan districts, Butajira city administration, South Central Ethiopia	Psychiatric nurses and health administrators or managers involved in co-ordinating and developing mental health care	A total of 28 (5 psychiatric nurses, 5 mental health co-ordinators from distri health offices, and 18 health centre heads or health centre focal persons for mental health).
Bengaluru, India	NIMHANS-tertiary care center	Psychiatry residents, trainees from M.Phil. in Psychiatric Social Work/Psychology/ M.Sc. Psychiatry nursing/staff who come in regular contact with person with mental illness (such as Instructors in Rehab departments etc.) from a tertiary care center.	24 mental health & non-mental health professionals
Delhi (National Capital Region), India	District Hospital in Faridabad, Haryana	Mental health professionals (1Psychiatrist, 1 Counsellor, 1 Psychiatric Nurse and 1 Community Nurse) from the district hospital	Four trained mental health professionals.
Kathmandu, Nepal	Pokhara, Gandaki Province	Psychiatry residents, psychiatrists, psychiatric nurses working in various private/government hospitals and medical colleges	6–8 psychiatrists, residents, and psychiatric nurses
Tunis, Tunisia	Razi University Hospital La Manouba	Psychiatry residents, nurses working in mental health at the hospital	16 – 20 psychiatry residents and nurses

**Table 2.** READ-MH training modules

Module	Module Title	Content / objectives	Teaching methods
	Introduction	Introduction of participants Introduction of Expert by Experience Learning objectives, module outline, course delivery methods	presentation
1	What is stigma and how does it affect mental health care?	Description and explanation of experienced, anticipated, self-stigma and structural stigma How stigma reduces effectiveness of mental health care	Interactive lecture Brainstorming Group discussion instigated by thought experiment and supported by evidence from the site Testimonial from expert by experience and/or audiovisual clip
2	Experienced discrimination	How to identify and respond to experienced discrimination as mental health professional How to help service users to respond to experienced discrimination How to develop stigma resistance in service users	Testimonial of expert by experience Role play and debriefing-group discussion
3	Anticipated discrimination and Self-stigma	How to identify and respond to anticipated discrimination as mental health professional How to help service users to overcome anticipated discrimination and self-stigma	Testimonial of expert by experience Case vignette Role play and debriefing-group discussion
4	Stigmatisation within mental health care	Structural stigma in mental health care Experienced stigma in mental health care (perpetrated by mental health professionals) Factors contributing to stigma perpetrated by mental health professionals	Interactive lecture Including quotes of stigmatising experiences from service users within the mental health system and clinical case illustrations Group discussion
5	What can we do to reduce stigma in mental health care?	Focus on stress and burnout as factors aggravating stigma; self-care for mental health professionals as a means to reduce stigma Communication skills Role of advocacy	Case study, followed by a Group discussion supported by evidence from the sites Role play

**Table 3.** Measures and assessment time points

	Baseline	During training	Immediate follow up	3-month follow up
Knowledge quiz	√	-	√	√
ASTAD	√	-	√	√
OSCE-rater	√	-	√	-
OCSE-simulated patient	√	-	√	-
Fidelity measure	-	√	-	-
Qualitative feedback	-	-	√	√

## Abbreviations

ASTAD: Attitudes to addressing stigma and discrimination scale
OSCE: Objective Structured Clinical Examination
READ-MH: Responding to Experienced and Anticipated Discrimination
